# Knowledge of Human Papillomavirus (HPV) and Oropharyngeal Cancer and Acceptability of the HPV Vaccine among Dental Students

**DOI:** 10.31557/APJCP.2020.21.12.3595

**Published:** 2020-12

**Authors:** Nada J Farsi, Sarah Al Sharif, Marwah Al Qathmi, Mazin Merdad, Hani Marzouki, Leena Merdad

**Affiliations:** 1 *Department of Dental Public Health, Faculty of Dentistry, King Abdulaziz University, Jeddah, King Saudi Arabia. *; 2 *Faculty of Medicine, King Abdulaziz University, Jeddah, King Saudi Arabia. *; 3 *Department of Otolaryngology, Head and Neck Surgery, King Abdulaziz University, Jeddah, King Saudi Arabia. *

**Keywords:** Human papillomavirus, oropharyngeal cancer, dental students, HPV vaccination

## Abstract

**Objectives:**

To assess knowledge about human papillomavirus (HPV), vaccination, and HPV-related oropharyngeal cancer (OPC) and to evaluate HPV vaccine acceptability among a sample of undergraduate dental students.

**Methods:**

All third- and fourth-year dental students enrolled in any of the dental schools in Jeddah, Saudi Arabia, were invited to participate in this cross-sectional study. In total, 500 students completed a self-administered questionnaire on HPV, HPV vaccines, and HPV-related OPC knowledge. HPV, HPV vaccine and HPV-related OPC knowledge scores were calculated. Associations of participants characteristics with the knowledge scores and with willingness to take the HPV vaccine were assessed.

**Results:**

Of the participants, 62% had heard of HPV infections, and 57% of HPV vaccines. Average knowledge scores were low: HPV knowledge score 3.8±4.5 out of 16, HPV vaccination knowledge score 0.9±1.6 out of 7, OPC knowledge score 0.9±1.2 out of 5. Clinical-year students had better knowledge and were more willing to take the HPV vaccine than were preclinical-year students, as were those vaccinated for hepatitis B versus those not vaccinated for hepatitis B. Students with higher HPV knowledge scores were more willing to take the HPV vaccine (66%) than were those with lower scores (43%) (p=0.018). Only 4% of males and 12% of females reported taking the HPV vaccine. Of those who refused it, 51% cited lack of knowledge as the primary reason.

**Conclusions:**

Knowledge about HPV, its vaccine, and HPV-related OPC is low among this sample of Saudi undergraduate dental students. Improving knowledge among them may increase their awareness, which could affect the care provided to patients.

## Introduction

Human papillomaviruses (HPVs) are sexually transmitted, and can cause infections in various body parts, including the oral cavity and oropharynx. There are more than 40 subtypes of HPV. High-risk HPV types, such as HPV 16 and 18, are etiological agents for cervical and anogenital tract cancers, as well as oropharyngeal cancers (OPC), a subset of head and neck squamous cell carcinomas (Centers for Disease Control and Prevention, 2019). A study in the United States estimated a 225% increase in the incidence of HPV-related OPC from 1988 to 2004; at that rate, the annual number of HPV-related OPCs would surpass cervical cancer by 2020 (Chaturvedi et al., 2011). OPC screening and HPV vaccination are important, as no specific diagnostic tool has been established for screening OPC that is equivalent to the Pap smear for cervical cancers (Al-Shaikh et al., 2014). HPV vaccines have demonstrated high effectiveness in preventing cervical and anogenital tract cancers and have the potential to reduce the incidence of OPCs as well (Chaturvedi et al., 2011; You et al., 2019). The growing prevalence of HPV-related OPC is becoming a major concern, as it is increasing regardless of the presence or absence of other OPC risk factors (older age, tobacco use, and excessive alcohol use) (Chaturvedi et al., 2011). 

Dentists can play a role in preventing HPV-related OPC by screening their patients for HPV-related OPCs (Macpherson et al., 2003; Daley et al., 2014) and by educating their patients about HPV and its risk factors, as well as encouraging them to get vaccinated. A study conducted among a sample of Florida dentists measured their willingness to discuss HPV vaccines with their patients and found that half of them (52%) fell into the precontemplation (not discussing and not intending) stage, followed by 40% of them in the contemplation (not discussing but willing to) stage, and only 9% in the maintenance (discussing with all patients) stage (Daley et al., 2014). Another study conducted in the United States found that dentists lacked the skills to address HPV-related issues with their patients, mainly because of the dentists’ young age, discomfort about discussing HPV topics, and lack of clarity about the role of dentists in discussing HPV knowledge and vaccines with patients (Daley et al., 2016). In addition, HPV knowledge among dental students was reported to be deficient by studies conducted in various countries, including the United States, Malaysia, and the Netherlands, and the need to implement educational material was highlighted (Daley et al., 2016; Poelman et al., 2017; Rajiah et al., 2017). Studies in the Middle East on HPV and vaccine knowledge among dental students are scarce. 

Acceptance of the HPV vaccine might be influenced by a number of enabling factors or barriers (Wong and Sam, 2010; Al-Shaikh et al., 2014). Several studies suggested that the cost of HPV vaccines is a major barrier, along with uncertainty about the side effects of the vaccine and the fear that daughters might engage in riskier sexual behavior after receiving it (Waller et al., 2006; Wong and Sam, 2010; Chiang et al., 2016; Hussain et al., 2016). In addition, the acceptability of the HPV vaccine was higher (7-fold increase) among female students than among non-students (such as employees), and the strongest predictor was a physician’s recommendation (93-fold increase) (Rosenthal et al., 2011). 

Given the mounting evidence on the association between HPV and OPC, it is crucial to determine dental student’s knowledge regarding HPV and HPV-related OPCs. Dental students are future healthcare providers who can help increase public awareness and promote HPV vaccination among their patients. To our knowledge, few studies examined dental students’ knowledge about HPV and the acceptability of the vaccine. The objectives of this study were (i) to determine knowledge about HPV and HPV vaccination, (ii) to evaluate knowledge about HPV-related OPCs, and (iii) to assess the acceptability of the HPV vaccine, among a sample of dental students.

## Materials and Methods

*Study Design and Participants*

This cross-sectional study was conducted in Jeddah, Saudi Arabia. The target population consisted of dental students enrolled during the academic year 2018-2019 in any dental school in Jeddah, Saudi Arabia. There are four dental schools in Jeddah; one is governmental, the Faculty of Dentistry at King Abdulaziz University (KAU), and three are private, Batterjee Medical College, Ibn Sina National College, and Alfarabi College. All dental students in these colleges in their third or fourth years were invited to participate in the study. Alfarabi College was excluded, as it did not have fourth-year students enrolled at that time. The reason for recruiting third- and fourth-year students is that they are still at an age when they can receive the HPV vaccine [11-26 years old according to Centers for Disease Control and Prevention, (2020) recommendations]. Third-year students are not exposed to clinical practice, and so we referred to them as “preclinical students,” whereas fourth-year students were referred to as “clinical students.” Ethical approval was obtained from the Ethical Committee at the Faculty of Dentistry at KAU (043-0318). Written permissions were obtained from each dental school, and visits were arranged with school administrators to collect the data. 

*Data Collection*

A self-administered questionnaire was distributed to eligible participants between March and October 2018. Two trained medical students distributed the questionnaires to the students in their classrooms after explaining the study objectives. Implied consent was obtained, as students were informed that filling in the questionnaire indicated their agreement to participate in the study. Participants were also informed that their participation was completely voluntary and that the anonymity and confidentiality of their responses would be preserved.

*Survey Instrument*

The questionnaire consisted of five main sections. The first section collected information about participants’ characteristics. The second and third sections included questions from a validated questionnaire that assessed general HPV and HPV vaccination knowledge (Waller et al., 2013). Sixteen general HPV knowledge questions were preceded by the question, “*Have you ever heard of HPV infection?*”, followed by a multiple-choice question about sources of HPV knowledge. Seven HPV vaccination knowledge questions were preceded by the question, “*Have you ever heard of the HPV vaccine?*”. The fourth section consisted of questions about HPV vaccine acceptability, including the following questions: “*Have you ever received an HPV vaccine?*”, “*Would you be interested in taking the HPV vaccine?*”, and “*What is the main reason you would not take the vaccine?*” (Centers for Disease Control and Prevention, National Center for Health Statistics, 2015-2016). Participants were also asked if an awareness program could help them decide whether to take the vaccine or not, and if they were willing to discuss the HPV vaccine with the same and with the opposite gender patients. The fifth section included knowledge questions about HPV-related OPCs (Rutkoski et al., 2017). The questionnaire was piloted on 20 fifth-year dental students to ensure clarity, practicability of the questions, and ease of completion. 

*Statistical Analysis*

The main outcome variables in this study were (i) HPV knowledge, (ii) HPV vaccination knowledge, (iii) HPV-related OPC knowledge, and (iv) HPV vaccine acceptability. A total knowledge score for HPV and HPV vaccination was calculated. The response to each knowledge question was given a score of 1 for a correct answer and 0 for “*I don’t know*” or an incorrect answer. For each participant, the total general HPV knowledge score and the HPV vaccination knowledge score were calculated by summing the scores of the responses in each corresponding section, with a final score ranging from 0 to 16 for HPV knowledge and from 0 to 7 for HPV vaccination knowledge. In addition, a total HPV-related OPC score was calculated for each participant by summing the scores of the responses to the OPC knowledge questions, with a final score ranging from 0 to 5. Lower scores indicated worse knowledge. 

Vaccine acceptability was categorized into a dichotomous variable, the categories being “Yes” and “No”/“I don’t know.” The associations between the following variables and vaccine acceptability were assessed: gender, academic year, cumulative grade point average (GPA), previous hepatitis B vaccination, previous history of sexually transmitted diseases (STDs), marital status, and the knowledge variables. 

Continuous variables were summarized by using means and standard deviations, and categorical variables were summarized by using frequencies and percentages. An independent sample t-test and a one-way analysis of variance were used to assess differences in mean values of the knowledge scores for categorical variables, as indicated. The chi-square test was used to assess the associations between categorical variables and vaccine acceptability. A p-value of ≤0.05 was considered statistically significant. SPSS Version 21 (SPSS Inc., Chicago, IL) was used for data analysis.

## Results

The questionnaire was completed by 500 out of 602 dental undergraduate students, yielding a response rate of 83%. The characteristics of the participants are summarized in Table 1. Sixty-five percent were female, 3% were married, and only 2% had children. Preclinical students represented 55% and clinical students 45% of the participants. Forty percent had an “excellent” cumulative GPA, 48% “very good,” and 12% “good and below.” Fifty-two percent of students reported being previously vaccinated for hepatitis B, and only 2% of participants had a previous history of STDs. Of the participants, 19% were current smokers and 70% had never smoked. Sixty-two percent of the students had heard of HPV infection, and 57% had heard of the HPV vaccine. 

Table 2 illustrates the associations between participants’ characteristics and their knowledge of HPV, the HPV vaccine, and HPV-related OPCs. The average HPV knowledge score was 3.8±4.5 out of 16, the average HPV vaccination knowledge score was 0.9±1.6 out of 7, and the average OPC knowledge score was 0.9±1.2 out of 5. Clinical-year students and those with previous hepatitis B vaccination had significantly higher knowledge scores than did preclinical students and those not vaccinated for hepatitis B (p<0.001). Female students had higher OPC knowledge (0.96±1.2) than male students did (0.7±1.1) (p=0.023). 

**Figure 1:**
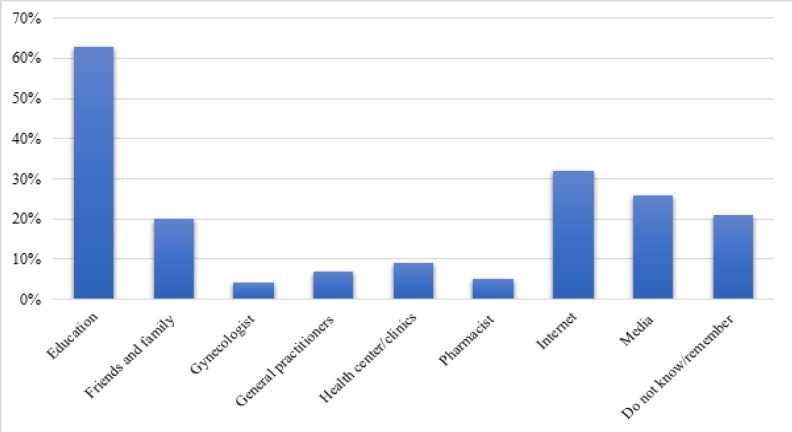
Dental Students’ Sources of Knowledge about Human Papillomavirus (HPV). Participants were asked about their sources only if they had heard of HPV

Figure 1 illustrates the sources of HPV knowledge for respondents who had previously heard of HPV. Education was the main source of information for 63% of students, followed by the Internet for 32%. Media was the main source for 26% of students, and friends and family for 20%. Only 4% of the participants stated that their gynecologist was their main source of knowledge.

Table 3 presents gender differences in acceptability of the HPV vaccine. Of the female students, 12% reported receiving the vaccine compared with 4% of the males (p=0.005). Around two-thirds of both women and men reported that vaccine awareness campaigns would benefit their decision to take the vaccine. Approximately 42% of the female students responded that they would discuss the HPV vaccine with female patients, and 31% of them would discuss it with male patients; for male students, 40% reported their willingness to discuss it with female patients and 36% with male patients. 

Associations between participants’ characteristics and their willingness to take the HPV vaccine are shown in Table 4. Clinical students were more willing to receive the HPV vaccine than were preclinical students, those who received the hepatitis B vaccine were more willing than were those who did not receive it, and those with high HPV knowledge scores were more willing than were those with low HPV knowledge scores. Other variables, including gender, marital status, cumulative GPA, history of STDs, HPV vaccine, and OPC knowledge scores, did not seem to affect a participant’s willingness to take the HPV vaccine.

**Figure 2:**
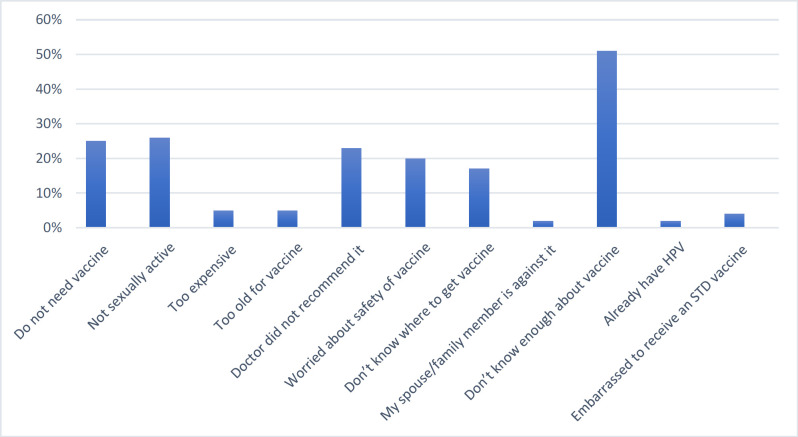
Reasons for Dental Students’ Lack of Willingness to Take the Human Papillomavirus (HPV) Vaccine. Participants were asked about their reasons only if they indicated that they did not want to be vaccinated

Figure 2 illustrates the reported reasons for unwillingness to take the HPV vaccine. The primary reason was students’ lack of knowledge about the vaccine (51%), followed by not being sexually active (26%). Twenty-five percent stated that they did not need the vaccine, and 23% that it had not been recommended by a doctor. Only 2% stated that a spouse/family member was against it. 

**Table 1:** Characteristics of the Participants

Variables	N=500 N (%)†
Academic year	
Preclinical	245 (55)
Clinical	201 (45)
Gender	
Male	167 (35)
Female	312 (65)
Marital status	
Single	478 (97)
Married	16 (3)
Children	
Yes	9 (2)
No	485 (98)
Cumulative GPA*	
Excellent	190 (40)
Very good	232 (48)
Good and below	57 (12)
Previously vaccinated with hepatitis B	
Yes	249 (52)
No	234 (48)
History of STDs	
Yes	10 (2)
No	482 (98)
Smoking status	
Current	95 (19)
Former	52 (11)
Never	344 (70)
Have you ever heard of HPV infection?	
Yes	307 (62)
No	186 (38)
Have you ever heard of the HPV vaccine?	
Yes	275 (57)
No	209 (43)

GPA, grade point average; STDs, sexually transmitted diseases; HPV, human papillomavirus; *GPA, excellent 4.50 – 5.00; very good 3.75 – 4.49; good and below 2.00 – 3.74; †, Some numbers do not add up to the total sample number because of missing values.

**Table 2:** Associations of Participant Characteristics with HPV, HPV Vaccination, and OPC Knowledge

Variables	HPV knowledge score (N=435)		HPV vaccination knowledge score (N=454)		HPV-related OPC knowledge score (N=446)	
	Mean (SD)	p-Value	Mean (SD)	p-Value	Mean (SD)	p-Value
Gender						
Male	3.4 (4.4)	0.129*	0.7 (1.5)	0.097*	0.7 (1.1)	0.023*
Female	4.1 (4.7)		1.0 (1.7)		0.96 (1.2)	
Academic year						
Preclinical	2.2 (3.6)	< 0.001*	0.3 (0.8)	< 0.001*	0.4 (1.0)	
Clinical	6.1 (4.8)		1.7 (1.97)		1.4 (1.3)	
Cumulative GPA‡	4.2 (4.8)		1.0 (1.6)		0.9 (1.1)	
Excellent	3.8 (4.5)		0.9 (1.6)		1.0 (1.2)	
Very good	3.1 (4.2)	0.353†	0.9 (1.5)	0.833†	0.9 (1.4)	0.915†
Good and below						
Previously vaccinated against hepatitis B		< 0.001*		< 0.001*		< 0.001*
Yes	4.7 (4.8)		1.3 (1.8)		1.1 (1.3)	
No	2.8 (4.1)		0.5 (1.3)		0.6 (1.1)	
Marital status						
Single	3.8 (4.5)	0.996*	0.9 (1.6)	0.911*	0.9 (1.2)	0.482*
Married	3.8 (5.3)		0.8 (1.7)		0.7 (1.0)	
STDs						
Yes	5.7 (5.3)	0.213*	1.9 (2.2)	0.082*	1.2 (1.3)	0.392*
No	3.8 (4.5)		0.9 (1.6)		0.9 (1.2)	
Smoking						
Current	3.6 (4.6)	0.582†	0.8 (1.5)	0.472†	0.8 (1.2)	0.253†
Former	3.2 (4.6)		1.1 (1.8)		1.1 (1.4)	
Never	3.9 (4.5)		0.9 (1.6)		0.8 (1.2)	
Total	3.8 (4.5)		0.9 (1.6)		0.9 (1.2)	

HPV, human papilloma virus; OPC, oropharyngeal cancer; GPA, grade point average; *, Two-sample t-test was used; †, One-way analysis of variance was used; ‡GPA, excellent 4.50 – 5.00, very good 3.75 – 4.49, good and below 2.00 – 3.74.

**Table 3:** Gender Differences in History of HPV Vaccination and Willingness to Recommend it to Patients

Variables	Male N=165 N (%)*	Female N=298 N (%)*	p-Value†
Have you ever received an HPV vaccine?			0.005
Yes	6 (4)	34 (12)	
No	151 (96)	255 (88)	
Do you think vaccine awareness programs would help you decide whether or not to take the vaccine?			0.5
Yes	102 (65)	197 (68)	
No	56 (35)	94 (32)	
Do you plan to discuss the HPV vaccine with your female patients?			0.671
Yes	66 (40)	126 (42)	
No	98 (60)	172 (58)	
Do you plan to discuss the HPV vaccine with your male patients?			0.218
Yes	60 (36)	91 (31)	
No	105 (64)	205 (69)	

HPV, human papillomavirus. *Some numbers do not add up to the total sample number because of missing values. †Chi-square test was used.

**Table 4:** Associations between Participant Characteristics and Willingness to Take the HPV Vaccine

Variables	Willing to take HPV vaccine N=216 N (%)*	Not willing to take HPV vaccine N=264 N (%)*	p-Value
Gender			
Male	70 (43)	92 (57)	0.417†
Female	141 (47)	158 (53)	
Academic year			
Preclinical	97 (41)	140 (59)	0.021†
Clinical	101 (52)	93 (48)	
Cumulative GPA§			0.471†
Excellent	82 (44)	106 (56)	
Very good	104 (47)	115 (53)	
Good and below	21 (39)	33 (61)	
Previously vaccinated against hepatitis B			0.007†
Yes	122 (51)	117 (49)	
No	87 (39)	138 (61)	
History of STDs			0.197‡
Yes	6 (67)	3 (33)	
No	205 (44)	259 (56)	
Marital status			0.886†
Single	206 (45)	254 (55)	
Married	6 (43)	8 (57)	
HPV knowledge score||			0.018†
High	19 (66)	10 (34)	
Low	168 (43)	223 (57)	
HPV vaccination knowledge score||			0.161†
High	17 (57)	13 (43)	
Low	181 (44)	235 (56)	
HPV-related OPC knowledge score||			0.905†
High	7 (44)	9 (56)	
Low	191 (45)	231 (55)	

HPV, human papillomavirus; GPA, grade point average; STDs, sexually transmitted diseases; OPC, oropharyngeal cancer; *Some numbers do not add up to the total sample number because of missing values. †Chi-square test was used; ‡Fisher’s exact test was used; §GPA, excellent 4.50 – 5.00; very good 3.75 – 4.49; good and below 2.00 – 3.74. ||, Knowledge score; 75% and above was considered high; below 75% was considered low.

## Discussion

Around two-thirds of the dental students in our study were aware of HPV infection. HPV and HPV-related OPC knowledge was significantly higher in clinical students than in preclinical students. Although 57% of students had heard of the HPV vaccine, only 9% reported being vaccinated. Overall, the knowledge of dental students regarding HPV vaccination was poor. The baseline knowledge of undergraduate students is an important area of emphasis because it will increase their contribution to primary prevention of HPV-related OPCs (Daley et al., 2014). 

The proportion of students who know of HPV infection varies widely in the literature. A recent study in Jordan found that 80% of the clinical dental students had heard of HPV infection (Sallam et al., 2019), whereas awareness of HPV was significantly lower among young female non-medical students targeted in Saudi (34.5%) and Nigeria (17.7%) (Makwe et al., 2012; Hussain et al., 2016). These findings highlight differences in the educational factor. In the current study, we noted that the lack of knowledge of HPV among dental students was high, which might be related to the low coverage that is given to HPV in educational programs in Saudi Arabia (Almutairi et al., 2019). Similar results have been reported among Indian dental students, where only 37% had heard of HPV infection (Saranya and Dhanraj, 2017).

Our results are similar to those of other studies in which differences in academic years influenced students’ HPV knowledge (Doshi et al., 2015; Poelman et al., 2017). Poelman et al., (2017) reported a significant difference in HPV knowledge between masters students (75.0%) and bachelor students (54.3%). Doshi et al., (2015) found that the mean total HPV knowledge score was higher among interns than among undergraduate dental students, which could be explained by the interns having completed their clinical training. Knowledge of HPV is directly related to academic level, with clinical-year students being more aware of the infection, probably due to the incorporation of theoretical information about HPV in their curriculum. No significant association was observed between GPA and the HPV knowledge score. Those who were previously vaccinated against hepatitis B had higher HPV knowledge scores. We believe that one of the important determinants in receiving the vaccination is knowledge; people aware of the hepatitis B vaccine may have more information about other infectious diseases such as HPV. Although our current study found no statistical difference in knowledge between men and women, the majority of female students answered correctly in regard to HPV knowledge. This finding accords with previously reported results. Almutairi et al., (2019) found that female medical students were more knowledgeable about it than males were (p<0.001), and Rajiah et al., (2017) suggested that the knowledge of their female dental participants had a direct relationship with women’s health, since they were receiving sufficient information about cervical cancer, Pap smear tests, and HPV. 

Our finding that education was the participants’ main source of information is similar to that of other studies from Malaysia (38.7%) and Saudi Arabia (45.1%) among medical students (Rashwan et al., 2012; Almutairi et al., 2019). This similarity underlines the importance of a university-based curriculum to address HPV knowledge. However, another study found that the media was the students’ main source of HPV knowledge (Rajiah et al., 2017). In our study, the Internet represented 32% of the responses and the media 26% of responses. This result demonstrates the potential for social media for developing effective educational material and interventions tailored to Saudi adolescents. 

The low vaccination rate of (4% among males, 12% among females) was similar to that in other studies, which reported a lower vaccination rate among medical Malaysian students (3.6%) and college students in Hong Kong (13.3%) (Rashwan et al., 2012; Chiang et al., 2016). This rate could be related to the lack of educational programs in schools, the uncertainty over side effects, and cultural boundaries. A review reported the estimated HPV vaccination coverage globally: 1.1% in Asia, 1.2% in Africa, 31.1% in Europe, and 35.6% in North America (Bruni et al., 2016). Many countries in these regions have added HPV vaccination to their national immunization schedules and raised public awareness about the usefulness of the vaccine in preventing some types of cancer.

Similarly, the effect was also reflected in HPV vaccine knowledge scores. In our study, the mean HPV knowledge score was 0.9 on a 7-item scale. This figure is similar to the results of previous studies that reported a low level of knowledge about HPV vaccination among university students in Hong Kong (Chiang et al., 2016) and Nigeria (Makwe et al., 2012). This figure contrasts, however, to that in an international comparison study, which reported higher knowledge about HPV vaccine, as measured on a 7-item scale, among United Kingdom (mean 3.8), United States (mean 3.9), and Australian populations (mean 4.0) (Marlow et al., 2013). In our study, 57% who were willing to take the vaccine had high HPV vaccine knowledge compared with 43% with high knowledge among those not willing to take the vaccine, although the difference was not statistically significant. It is striking that undergraduate dental students were insufficiently knowledgeable about HPV vaccination; this finding highlights the need for future efforts to raise awareness. As Chiang et al., (2016) reported, vaccination uptake is directly correlated with students’ baseline knowledge about HPV vaccination.

Forty-five percent of the study participants intended to take the HPV vaccine, a relatively low rate compared with other studies among university and dental students, in which the rate ranged between 48% and 81.7% (Daley et al., 2010; Wong and Sam, 2010; Chiang et al., 2016; Rajiah et al., 2017). Our participants’ low knowledge of HPV vaccination could explain the relatively low rate of intention to take the vaccine. In addition, Saudi society is religious and conservative, and sexually transmitted infections are less likely to occur in the absence of sexual behavior before marriage. Nonetheless, Rajiah et al. (2017) reported that 90% of their dental students would be vaccinated if they had the information. Therefore, knowledge about the vaccine is essential for students to decide about vaccination. In our study, 52% of the clinical students were willing to be vaccinated compared with 41% of the preclinical students. Sixty-six percent of those who scored high in HPV knowledge were willing to be vaccinated, whereas 57% who scored low were not willing. 

The current study also sheds light on the unwillingness of the participants (55%, data not shown) to receive the HPV vaccine. Because of the conservative culture of the Saudi population, the sexual nature of HPV infection might introduce barriers to HPV information seeking, vaccination education, and awareness, such as the fear of being labeled sexually active. Of our participants, 23% reported that HPV vaccination had not been discussed or recommended by a healthcare provider, and 20% worried about the safety of the vaccine. These findings are consistent with others: hence the need for strategies at a regional level, as well as community partnerships, constructed carefully so that women feel less stigmatized (Wong and Sam, 2010; Rosenthal et al., 2011; Chiang et al., 2016).

Gender was a barrier to discussing HPV with patients. Participants expressed more hesitation in relation to discussing or recommending HPV vaccines with the opposite gender, yet the differences were not statistically significant. The low rate of willingness to address sex-related topics could be related to factors such as education, cultural barriers, religious factors, and individual beliefs (Poelman et al., 2017). In a study conducted among Florida dentists, 44% of female dentists were in the action stage (recommending the vaccine) (Daley et al., 2014). A study of Dutch dental students found that they thought dentists should discuss HPV vaccination with their patients (Poelman et al., 2017). Findings suggest that dentists have an important role in encouraging HPV vaccination (Daley et al., 2010). In our study, 65% of the male students and 68% of the female students thought that public vaccination awareness campaigns would help them to take the vaccine. 

The growing burden of HPV-related OPCs highlights the need for dental providers to play a role in this emerging public health problem. HPV-related OPC prevalence increases across calendar periods: hence the need to focus on prophylactic HPV vaccination (Chaturvedi et al., 2011; Centers for Disease Control and Prevention, 2019). The incidence of OPC in Saudi Arabia is 2.4% and that of cervical cancer is 1.4% (Ferlay et al., 2012). A recent study that evaluated the prevalence of HPV with head and neck cancer showed an increase in OPCs over the years, 21% being associated with HPV infection (Alsbeih et al., 2019). 

Macpherson et al., (2003) reported that dental practitioners were more confident in identifying potential precancerous lesions than were medical practitioners. Nonetheless, in our study, participants’ overall knowledge about OPC was inadequate (mean 0.9±1.2 out of 5). Only 29% affirmed that there is a link between HPV infection and OPCs (data not shown). Studies showed higher knowledge in linking HPV and OPCs in Dutch (64%) and Spanish (75%) dental students (Poelman et al., 2017; Lorenzo-Pouso et al., 2018). A study conducted in Saudi Arabia by Kujan et al., (2014) among all dental students in their fourth to sixth years that assessed oral cancer knowledge showed that 90% of dental students identified smoking, 87% identified alcohol, and 84% identified HPV as risk factors. Kujan et al., (2014) reported higher scores for those with a higher level of university education who had some exposure to oral cancer during their clinical placement. In our results, 48% of clinical students linked HPV with OPCs, compared with only 10% of preclinical students (data not shown). This finding highlights the importance of knowledge in identifying HPV as a risk factor for OPC. Another factor could be that clinical students have higher confidence because they have more knowledge and experience (Lorenzo-Pouso et al., 2018). Female dental students demonstrated better HPV-related OPC knowledge than did their male counterparts, and the difference was significant. These findings are aligned with those of an earlier report (Rajiah et al., 2017), suggesting that female students may gain their knowledge from cervical cancer awareness programs. Despite the literature reporting that HPV-related OPCs develop more in males, they scored low in our study (Rutkoski et al., 2017). 

The limitation of this study is that women were overrepresented among our participants, signaling the potential for possible selection bias. Another limitation is that the long-term effect of the students’ education could not be tested, as we targeted participants who were still young enough to receive HPV vaccination according to the CDC’s recommendation (Centers for Disease Control and Prevention, 2020). Despite these limitations, our study had a high response rate. Given the sampling technique (targeting all dental schools in Jeddah) and the high response rate, these results should be representative of the dental students of the specified age in the dental schools in Jeddah. However, caution should be practiced when generalizing the results to other dental schools. In addition, previous studies conducted in Saudi Arabia on HPV were within the context of cervical cancer and mostly among women. Our study targeted both genders and inquired about overall knowledge of HPV, vaccination, and its relation to OPC, due to the growing prevalence of HPV and HPV-related OPCs. 

In conclusion, overall knowledge about HPV, the HPV vaccine, and HPV-related OPCs is deficient among this sample of Saudi undergraduate dental students. Those in their clinical year were more knowledgeable about HPV. More than half of them reported willingness to take the vaccine. Targeting providers’ education programs and addressing the gaps in dental students’ knowledge may increase the students’ willingness to raise public health awareness and recommend HPV vaccination. Further assessment of student knowledge after implementation of an educational curriculum is warranted.
